# Identification and Characterization of New Molecular Partners for the Protein Arginine Methyltransferase 6 (PRMT6)

**DOI:** 10.1371/journal.pone.0053750

**Published:** 2013-01-10

**Authors:** Alessandra Lo Sardo, Sandro Altamura, Silvia Pegoraro, Elisa Maurizio, Riccardo Sgarra, Guidalberto Manfioletti

**Affiliations:** Department of Life Sciences, University of Trieste, Trieste, Italy; University of Melbourne, Australia

## Abstract

PRMT6 is a protein arginine methyltransferase that has been implicated in transcriptional regulation, DNA repair, and human immunodeficiency virus pathogenesis. Only few substrates of this enzyme are known and therefore its cellular role is not well understood. To identify in an unbiased manner substrates and potential regulators of PRMT6 we have used a yeast two-hybrid approach. We identified 36 new putative partners for PRMT6 and we validated the interaction *in vivo* for 7 of them. In addition, using *in* *vitro* methylation assay we identified 4 new substrates for PRMT6, extending the involvement of this enzyme to other cellular processes beyond its well-established role in gene expression regulation. Holistic approaches create molecular connections that allow to test functional hypotheses. The assembly of PRMT6 protein network allowed us to formulate functional hypotheses which led to the discovery of new molecular partners for the architectural transcription factor HMGA1a, a known substrate for PRMT6, and to provide evidences for a modulatory role of HMGA1a on the methyltransferase activity of PRMT6.

## Introduction

Protein arginine methylation is a post-translational modification (PTM) that has been implicated in a large variety of important cellular functions such as signalling, DNA repair, RNA maturation and nucleocytoplasmic transport, protein protection, ribosomal assembly, and regulation of gene expression [1]. In mammalian cells arginine methylation is performed by a sequence-related family of protein arginine methyl transferases termed PRMTs. Given that this family of enzymes plays an integral role in many cellular processes, it is unsurprising that their dysregulation is involved in several human diseases [1]–[4]. Currently, nine different human PRMTs are known (PRMT1-9). PRMTs share a set of conserved sequence motifs and a THW (threonine-histidine-tryptophan) loop, but differ in the presence of additional protein domains, cellular localization, and tissue expression. There does not appear to be major redundancy between these enzymes since mouse knockouts display generally clear and dramatic phenotypes [1].

PRMT6 is a predominantly nuclear enzyme characterized by substrates specificity and by automethylation [5]. In particular, PRMT6 is the major PRMT responsible for histone H3R2 methylation and it has a clear role in antagonizing the Mixed-Lineage Leukaemia (MLL)-complex-dependent methylation of the Lys-4 residue [6]–[8]. Methylation of H2AR29 is specifically enriched at genes repressed by PRMT6, implicating also this modification in transcriptional repression [9]. In addition PRMT6 binds and methylates the architectural transcription factor HMGA1 [10], [11]. These evidences underline an important function for this enzyme in the context of chromatin structure organization and epigenetic regulation. PRMT6 has shown to directly impact transcription, in fact thrombospondin-1 (TSP-1) was identified as a transcriptional repression target of PRMT6 by directly regulating the TSP-1 promoter activity [12]. Further involvement of PRMT6 in regulation of gene expression is provided by the coactivation of progesterone, glucocorticoid and oestrogen receptors [13]. Besides the involvement of PRMT6 in epigenetic and transcription, arginine methylation by PRMT6 was shown to have a negative impact on the activities of HIV-1 Tat, Rev and nucleocapsid proteins, thus potentially affecting HIV replication [14]–[17]. In addition, PRMT6 was demonstrated to specifically methylate DNA polymerase ß resulting in a strong stimulation of DNA polymerase activity by enhancing DNA binding and processivity, thus involving PRMT6 in base excision repair (BER) [18].

Very recently, PRMT6 was found to be involved in the control of cell cycle progression repressing key cell-cycle regulators, i.e. p21 (CDKN1a), p27 (CDKN1B), and p16 (CDKN2A) [19]–[21]. For both p21 and p27, repression is concomitant with the presence of PRMT6-dependent H3R2 methylation at the promoter level [19], [20]. The same mechanism of repression has been demonstrated towards p53 [22], clearly linking PRMT6 with the process of tumorigenesis.

The rapidly growing importance of PRMT6 highlights the need to investigate more deeply the mechanisms this enzyme is involved in.

We adopted the yeast-two hybrid (Y2H) assay to obtain a protein network providing a more detailed picture of the molecular context in which PRMT6 is embedded. Moreover, we demonstrated the utility of this molecular network in supporting the set-up of hypothesis-driven experiments. Indeed, both new connections and modulatory mechanisms within the PRMT6 molecular network have been highlighted.

## Materials and Methods

### Plasmids

Plasmids pEG202 PRMT6/1-86,/1-184,/87-184,/87-375, and/185-375, expressing deletion mutants of human PRMT6 protein, were generated by PCR using the human PRMT6 cDNA as a template as described in Materials and Methods S1. The human fetal brain cDNA library in the pJG4-5 plasmid was a kind gift of Dr. G. Del Sal. pGEX4T-2 HMGA1b, pGEX4T-2 HMGA2, pGEX-6P-1 PRMT6, and pGEX2T-GAR have been described previously [23], [5], [24]. Each putative partner was subcloned between the *Eco*RI and *Xho*I sites of the vectors pcDNA3HA and pGEX4T-1. The mammalian expression vector pcDNA3MBP-PRMT6 was generated by subcloning PRMT6 from pGEX6P-1 PRMT6 in the *Bam*HI and *Xho*I sites of pcDNA3MBP (kindly provided by Dr. L. Collavin). All the tags are in fusion with the N-terminal portion of the proteins. Plasmids pARHMGA1a, pARHMGA1a-(1–52), and pARHMGA1aR57,59A for the expression of wild-type and mutant forms of human HMGA1a proteins were previously described [11].

### Yeast Two-hybrid Screening

pEG202-PRMT6 vector was used to express wild type PRMT6 linked to the C-terminus of LexA DNA-binding domain. Recombinant plasmid was transfected in *Saccaromyces cerevisiae* strain EGY48 (MATα, Trp1, Ura3, His3, LEU2::LexAop6LEU2). A human fetal brain cDNA library expressing Gal4 activation domain fusion proteins was transfected in the yeast strain containing pEG202-PRMT6 for a screening assay. A total of ∼6.4×10^6^ transformants were plated on Complete Minimal (CM) medium supplemented with galactose/raffinose, lacking histidine, tryptophan, uracile and leucine. After 4 days, clones were tested by a β-galactosidase filter assay. Among these, 360 were tested in a secondary screening. For this purpose the plasmid coding for each putative partner was extracted and used to transform *E. coli* strain B290 with electroporation. For each transformation 3 different colonies were analyzed and positive plasmids were re-transformed into EGY48 with pEG202-PRMT6; 179 clones were confirmed to be Leu^+^ and LacZ^+^. The DNA inserts of the library plasmids were sequenced and 36 clones resulted to be unique.

To map the interacting domain in PRMT6, the yeast strain EGY48 was transfected with pEG202-PRMT6/1-86,/1-184,/87-184,/87-375, and/185-375 and each clone was transfected with the partner cloned in pJG4-5. Clones were assayed for the interaction as above described.

### GST Pull-down assay, Co-Affinity Purification and Western-blot Analysis

Expression, purification of GST fusion proteins, GST pull-down assays, co-affinity purification (co-AP) experiments, and western blot analyses were performed by standard procedures as previously described [23], [25]. As assed by densitometric analyses of Blue Comassie stained gels, the purity of the GST-fused proteins was about 50% for GST-HMGA2, 80% for GST-HMGA1b and 60% for PRMT6. Antibodies used in western blots were α-LexA (Santa Cruz) and α-HA (Sigma).

### In Vitro Methylation Assay

GST-PRMT6, -NOB1, -HSJ-2, -MIF, -Nm23-H1, -SVEP1, -TUBB2A, -snRNPB, and -GAR proteins were expressed and purified as describe above. GST-fused proteins were eluted using standard procedures and analysed for correct production and quantification by SDS-PAGE (T = 10%) and Blue Comassie staining. 2 µg of each putative partner was incubated with 0.5 µg of recombinant GST-PRMT6 protein in presence of S-adenosyl-L-(methyl-^3^H)-methionine (Perkin Elmer) for 1 hour at 30°C. The reaction was stopped by heat inactivation (5 minutes at 94°C) in SDS sample buffer. Proteins were then separated by SDS-PAGE (T = 10%). Gels were stained by Blue Comassie, soaked in NAMP-100 Amplify (Amersham Biosciences), dried, and exposed to Hyperfilm MP (GE Healthcare) at −80°C for few days. Methylation assays of MIF in the presence of recombinant HMGA1a proteins forms (wild-type, 1–51, and R57,59A HMGA1a) were performed as described above. Production of recombinant HMGA1 proteins has been previously described [11].

## Results and Discussion

### New Molecular Partners for PRMT6: a Yeast Two-hybrid Screening

A Y2H screening with full length PRMT6 (aa 1–375) as bait was performed to identify putative interaction partners and hopefully potential substrates of PRMT6. 179 clones were confirmed and their cDNA has been sequenced. Among these, 36 resulted to be unique and are listed in Table S1. Bioinformatic analyses (DAVID - Database for Annotation, Visualization and Integrated Discovery bioinformatic tools) evidenced that all the identified partners are intracellular, a consistent number (21) has a predominant nuclear localization, which is consistent with PRMT6 localization [5], and that most of them (27) are localized in the cytoplasm with a considerable number (13) associated with intracellular non-membrane-bounded organelle (Table S2). From the gene ontology (GO) annotations of the identified partners, PRMT6 appears to be involved in protein complex assembly, RNA processing, and regulation of protein metabolic processes, in particular nucleoside and nucleotide metabolic processes. None of the proteins listed have previously been identified as partners or substrates of PRMT6.

Some of the identified PRMT6’s partners support the reliability of our Y2H screening. hnRNP Q and snRNPB are already known to be substrates for PRMTs. Med28 and MTF2 are subunits of macromolecular complexes involved in gene transcription and chromatin epigenetic modulation, activities that have been firmly established also for PRMT6 [6]–[9].

hnRNP Q has been previously shown to be methylated *in vitro* by PRMT1 and its *in vivo* methylation is important for its nuclear localization [26] and for insulin receptor trafficking and insulin signalling [27]. The small nuclear ribonucleoprotein B and B1 (snRNPB), which is involved in several steps of the biogenesis of the snRNPs, has also been found methylated on arginine residues but the PRMT responsible for this modification has not been identified yet [28]. Med28 is a subunit of Mediator, a transcriptional cofactor that regulates transcription of nearly all RNA polymerase II-dependent genes [29] while MTF2 is a member of polycomb repressive complex-2 (PRC2) that is involved in the epigenetic regulation of a large number of genes [30].

### Mapping the PRMT6’s Protein-protein Interaction Domain

**Figure 1:**
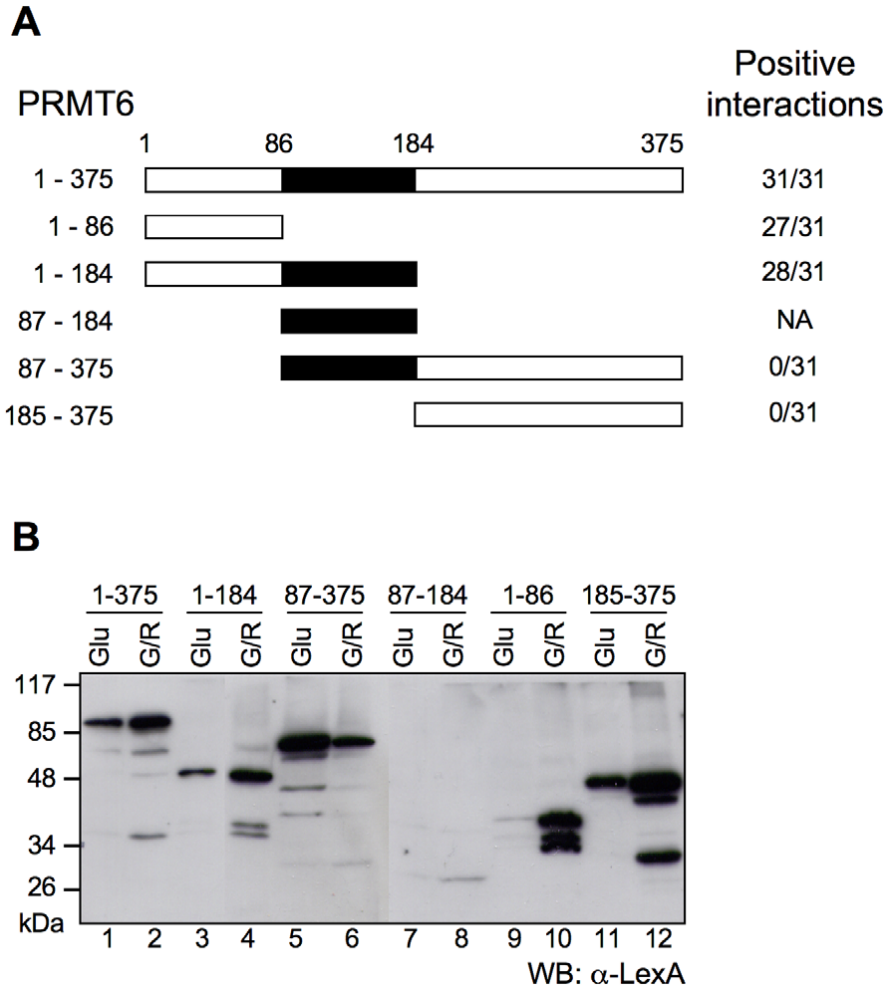
PRMT6 protein-protein interaction domain maps mainly to the N-terminal protein portion 1–86. (A) Schematic representation of the PRMT6 deletion mutants. The central region containing the catalytic domain is indicated in black. Numbers correspond to the aminoacids positions. Positive interactions out of 31 partners tested are indicated on the right. (B) Total protein extracts from yeast expressing the different PRMT6 deletion mutants in fusion with LexA DNA binding domain, grown in medium containing glucose (Glu) or galactose and raffinose (Gal/Raf), were separated in SDS-PAGE (T = 10%) and analyzed by western blot using an α-LexA antibody. Molecular weights are indicated on the left.

Little information is available about the domain organization of PRMT6 and PRMTs in general, therefore the region of PRMT6 involved in the interaction with the putative partners was mapped. To this end five different N- and C-terminal deletion mutants of PRMT6 were generated (Fig. 1A). Before testing the interaction in Y2H assay, their expression in yeast was assessed by western blot. They were all correctly expressed except mutant PRMT6 87–184 (Fig. 1B). Each PRMT6 deletion mutant was tested for the interaction with a subset of 31 partners out of the 36 identified. Full length PRMT6 and ornitine decarboxylase (ODC) were included as positive and negative control, respectively. Results reported in Table S3 and summarized in Fig. 1A show that 27 out of 31 partners tested are able to interact with the deletion mutant PRMT6 1–86, while none of them is able to bind mutants lacking the N-terminal portion of PRMT6, suggesting therefore that the N-terminal region is necessary and sufficient for the association with the partners. It is worthwhile noting that, in a first attempt to clone PRMT6 a cDNA was isolated encoding for a 60 aa N-terminal truncated form of PRMT6 that was missing methyltransferase activity [5], therefore suggesting an involvement of this region in substrate docking.

Further evidences suggest a role for the N-terminal region of PRMTs in substrate binding specificity and enzymatic activity. Indeed, it has been demonstrated that alternative splicing of PRMT1 generates several N-terminal isoforms differing in catalytic activity and substrate specificity [31] and that N-terminal domain of PRMT8 modulates its activity [32].

These observations are supported by data evidencing that within PRMT1 substrates, positively charged residues distal to the modified arginine are involved in the process of docking to the enzyme surface [33]. In agreement with this, a surface scanning mutational analysis of PRMT1 revealed that mutation of N-terminal acidic residues within the EEMxxD motif strongly impairs substrate binding [34]. It is noteworthy to evidence that this motif is perfectly conserved between PRMT1 and PRMT6.

Previously structural work on PRMT1, 3 and 5 [35]–[37] suggested a role for the N-terminal portion of PRMTs in protein-protein interactions and the possibility that the sequence variability of PRMTs could be responsible for their different substrate specificity.

All these structural and biochemical data suggest a key role of the N-terminal portion of PRMTs in substrate binding and are in agreement with our data demonstrating that the N-terminal portion of PRMT6 (aa 1–86) is essential for binding to its molecular partners.

### In Vitro and In Vivo Orthogonal Confirmation of PRMT6’s Molecular Partners

**Figure 2:**
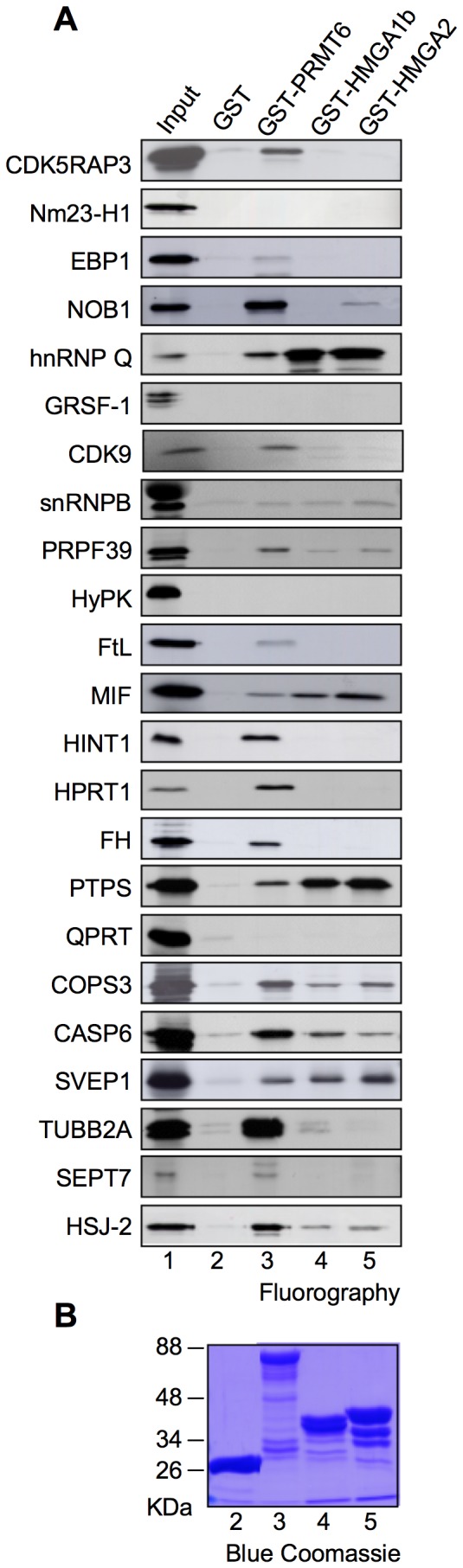
Confirming PRMT6’s partners and extending HMGA1a molecular network by a hypothesis driven approach. (A) Putative PRMT6 molecular partners were *in vitro* translated (IVT) and radiolabelled by incorporation of ^35^S-methionine. GST pull-down assays were performed with GST, GST-PRMT6, GST-HMGA1b, and GST-HMGA2. For each partner, in lane 1 (input) a quantity corresponding to 20% of the IVT used in the GST pull-down experiments was loaded. Proteins are visualized by fluorography. Experiments were repeated at least twice and a representative result is shown. (B) A representative SDS-PAGE (T = 10%) of the proteins used in the experiments, stained with Blue Comassie, is shown.

To confirm the interactions found, we performed *in vitro* pull-down assays using recombinant GST-PRMT6 and *in vitro*-translated HA-tagged PRMT6’s partners. Among the 31 partners, 23 were tested in GST pull-down assays since 8 of them (Med28, MTF2, UTP6, PRDX4, SAAL1, L38m, LDH-B and PRKX) were not efficiently *in vitro* translated (data not shown). As shown in Fig. 2, the interaction with PRMT6 was confirmed for 19 out of the 23 partners tested. 4 proteins (Nm23-H1, GRSF-1, HYPK and QPRT) were not confirmed, probably because the interaction in yeast was not direct, or because PTMs could be needed for the interaction.

We previously demonstrated that PRMT6 is able to both associate and methylate HMGA1a [11], which is a nuclear architectural factor able to coordinate the assembly of DNA-bound macromolecular complexes regulating transcription [38]. Within these complexes HMGA proteins establish multiple protein-protein contacts. Co-activators (or co-repressors), such as the acetyltransferases CREB binding protein/p300 (CBP/p300) and CBP/p300-associated factor (P/CAF), are often integral components of these complexes that modify the chromatin structure through PTMs of histone tails.

Since PRMT6 is a HMGA interactor and a histone modifier that can be part of DNA-bound complexes, we hypothesized that HMGA and PRMT6 could have overlapping molecular contexts and common partners. Therefore, PRMT6 partners were assayed in GST-pull down experiments for their interaction with two HMGA proteins: HMGA1b (the shorter isoform of HMGA1 proteins), and HMGA2. HMGA1a isoform turned out not to be efficiently produced as a GST-fusion product (data not shown). In agreement with our hypothesis, among the 19 confirmed PRMT6’s interactors, 9 were found to interact with both HMGA1b and HMGA2 (hnRNP Q, snRNPB, PRPF39, MIF, PTPS, COPS3, CASP6, SVEP1 and HSJ-2) and 1 protein (RNA binding protein NOB1) resulted to specifically interact only with HMGA2.

**Figure 3:**
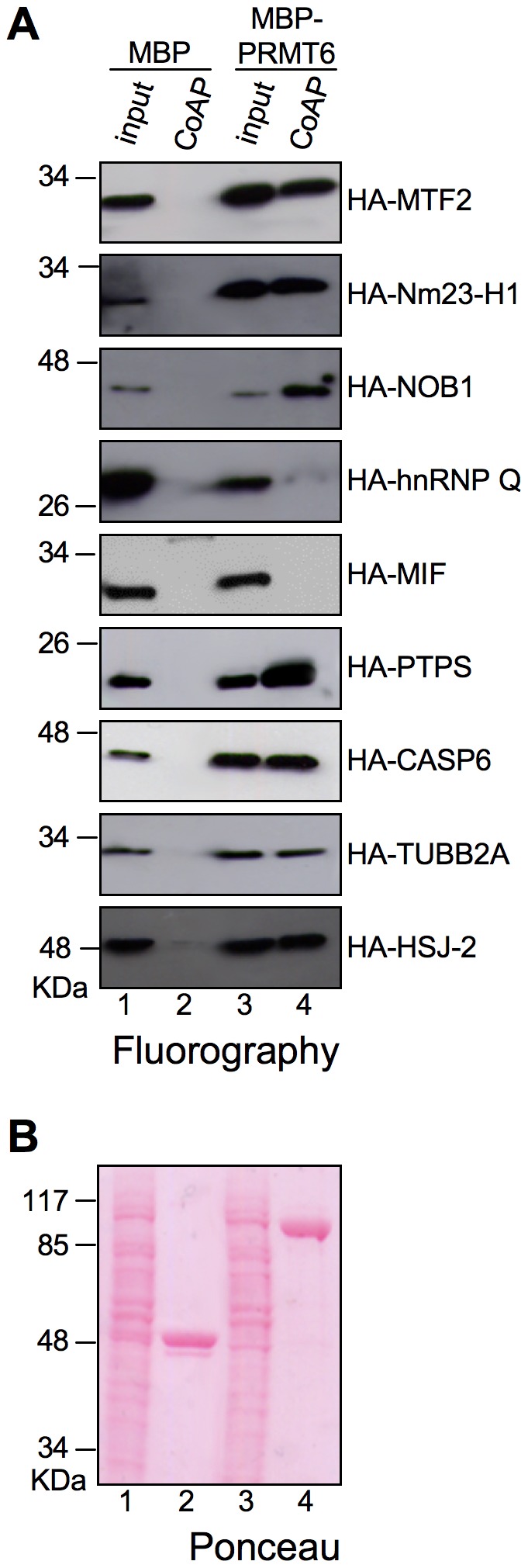
In vivo confirming PRMT6’s partners by Co-Affinity purification (Co-AP). (A) PRMT6 fused to Maltose Binding Protein (MBP-PRMT6) or Maltose Binding Protein (MBP) alone were produced by transient transfection in HEK293T cells. Cellular lysates were incubated with amylose resin and affinity captured MBP-PRMT6 and MBP proteins recovered. Proteins were separated by SDS-PAGE (T = 10%) and analysed by WB using an α-HA antibody. Lanes 1 and 3: input, 5% of the amount used; lanes 2 and 4: co-affinity purified proteins. Experiments were repeated at least twice and a representative result is shown. (B) The ponceau stained membrane of a representative experiment is shown.

To further validate our screening, 9 partners were selected to be assayed *in vivo* for their ability to bind PRMT6 using co-Affinity Purification (co-AP). PRMT6 was cloned in fusion with the Maltose Binding protein (MBP) and co-transfected in cells together with partners cloned in fusion with HA tag. Cells were lysed in native conditions, the complexes were purified with the amylase resin, analysed by SDS PAGE, and the partners detected by western blot analyses using α-HA antibody. Also in this case, the vast majority of the tested proteins turned out to be confirmed. Indeed, Fig. 3 shows that among the partners tested, 7 (MTF2, Nm23-H1, NOB1, PTPS, CASP6, TUBB2A, and HSJ-2) resulted to interact with PRMT6 while only 2 (hnRNP Q and MIF) were not confirmed.

### Identifying New Substrates for PRMT6 among its Molecular Partners

**Figure 4:**
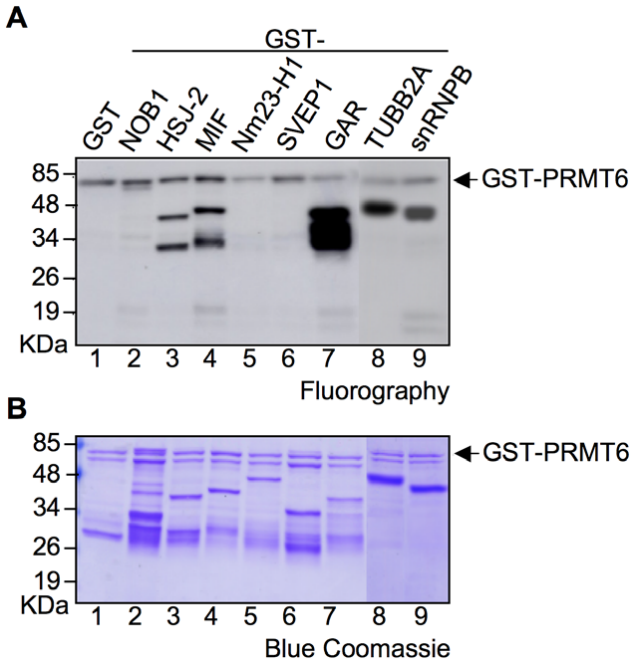
Discovering new substrates for PRMT6. (A) Recombinant PRMT6 partners in fusion with GST were incubated with radio-labelled S-adenosyl-L-(methyl-^3^H)-methionine and GST-PRMT6 for *in vitro* methylation assay (lanes 2–6, 8, 9). GST and GST-GAR (lanes 1 and 7) were used as negative and positive control, respectively. Proteins were separated by SDS-PAGE (T = 10%) and checked by fluorography. Experiments were repeated at least twice and a representative result is shown. (B) Blue Comassie staining was used to check both the correct production and the amount of recombinant proteins. Arrows indicate the position of PRMT6.

To determine whether the identified partners could be new substrates of PRMT6 we performed *in vitro* protein methylation assays (Fig. 4). We assayed 5 proteins that were confirmed *in vivo* as PRMT6 partners (Nm23-H1, HSJ-2, SVEP1, NOB1 and TUBB2A) and 2 factors that were in common with both PRMT6 and HMGA (snRNPB and MIF). Recombinant proteins in fusion with GST were incubated in the presence of [^3^H]-AdoMet (*S*-adenosyl-L-[*methyl*-^3^H]-methionine) with GST-PRMT6. GST and GST-GAR were used as negative and positive control respectively and in fact a strong signal was detected for GST-GAR (lane 7) while no signal (except that corresponding to automethylation of PRMT6) is present for GST (lane 1). HSJ-2, MIF, TUBB2A and snRNPB proven to be efficiently methylated by PRMT6 (Fig. 4 - lanes 3, 4, 8 and 9); noteworthy, all these 4 proteins contain an RGG/RXR motif which is considered the consensus recognized by PRMTs [1], [2], [10], [11].

### From Overlapping Molecular Networks to Functional Hints

Proteins embedded in the same molecular network and in close proximity to each other should be somehow functionally linked. As a proof of concept of the validity of this assumption, we evaluated whether there could be a functional link between HMGA, PRMT6, and MIF. Indeed, these three proteins are linked each other by direct protein-protein interactions and PRMT6 is a bona fide modifying enzyme for the other two. Moreover, MIF and HMGA1 are functionally linked since both have been demonstrated to interact with p53 and to be involved in the negative modulation of p53 activity [39]–[41].

We performed a methylation assay in the presence of constant amount of MIF and increasing concentration of HMGA1a in order to evaluate a modulatory role of HMGA1a with respect to the methylation activity of PRMT6 toward MIF. A truncated HMGA1a form (1–51) and a R57,59A double mutant were also included in the assay. The 1–51 HMGA1a truncated form is not able to bind PRMT6 and is not methylated by PRMT6 [11] while the R57,59A mutant is still able to bind PRMT6 (our unpublished data) but is modified by PRMT6 much less efficiently at minor methylation sites [11].

**Figure 5:**
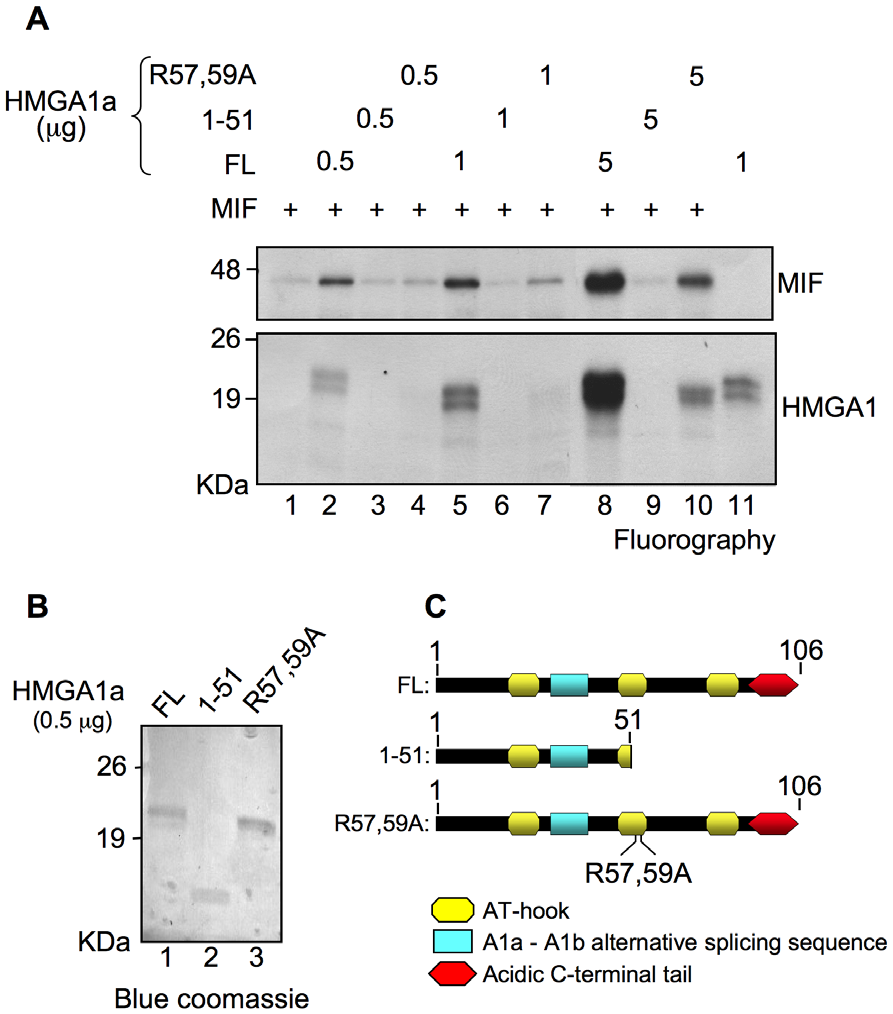
HMGA1a modulates the methyltransferase activity of PRMT6 toward MIF. (A) Constant amount of GST-MIF were incubated with recombinant GST-PRMT6 and radio-labelled S-adenosyl-L-(methyl-^3^H) methionine in the presence of increasing amounts of full length HMGA1 (FL), a truncated HMGA1a form (1–51), and a HMGA1a mutated form (R57,59A) for *in vitro* methylation assays. Methylation reactions of MIF and HMGA1a alone represent control experiments. Proteins were separated by SDS-PAGE (T = 15%) and checked by fluorography. Experiments were repeated at least twice and a representative result is shown. (B) Blue Comassie staining was used to check for the quantification of HMGA1a proteins. (C) Schematic representation of the FL, 1–51 and R57,59A HMGA1a domain organization.

As it is possible to see from Fig. 5, the presence of a wild-type HMGA1, which is able both to interact with and to be methylated by PRMT6, strongly enhances the PRMT6-dependent methylation of MIF; on the contrary, neither the presence of a truncated HMGA1a nor that of a not-methylatable protein exerts a relevant effect with respect to the methylation efficiency of PRMT6 towards MIF. Since the R57,59A mutant is still able to bind PRMT6, the modulatory role of HMGA1a towards PRMT6 activity seems to be linked to HMGA1a R57,59 methylation status.

The possibility that interacting partners of PRMT6 could modulate the processivity of this enzyme was already envisioned [42]. Further experiments will be needed to clarify the mechanism responsible for the modulatory role of HMGA1 towards PRMT6.

**Figure 6:**
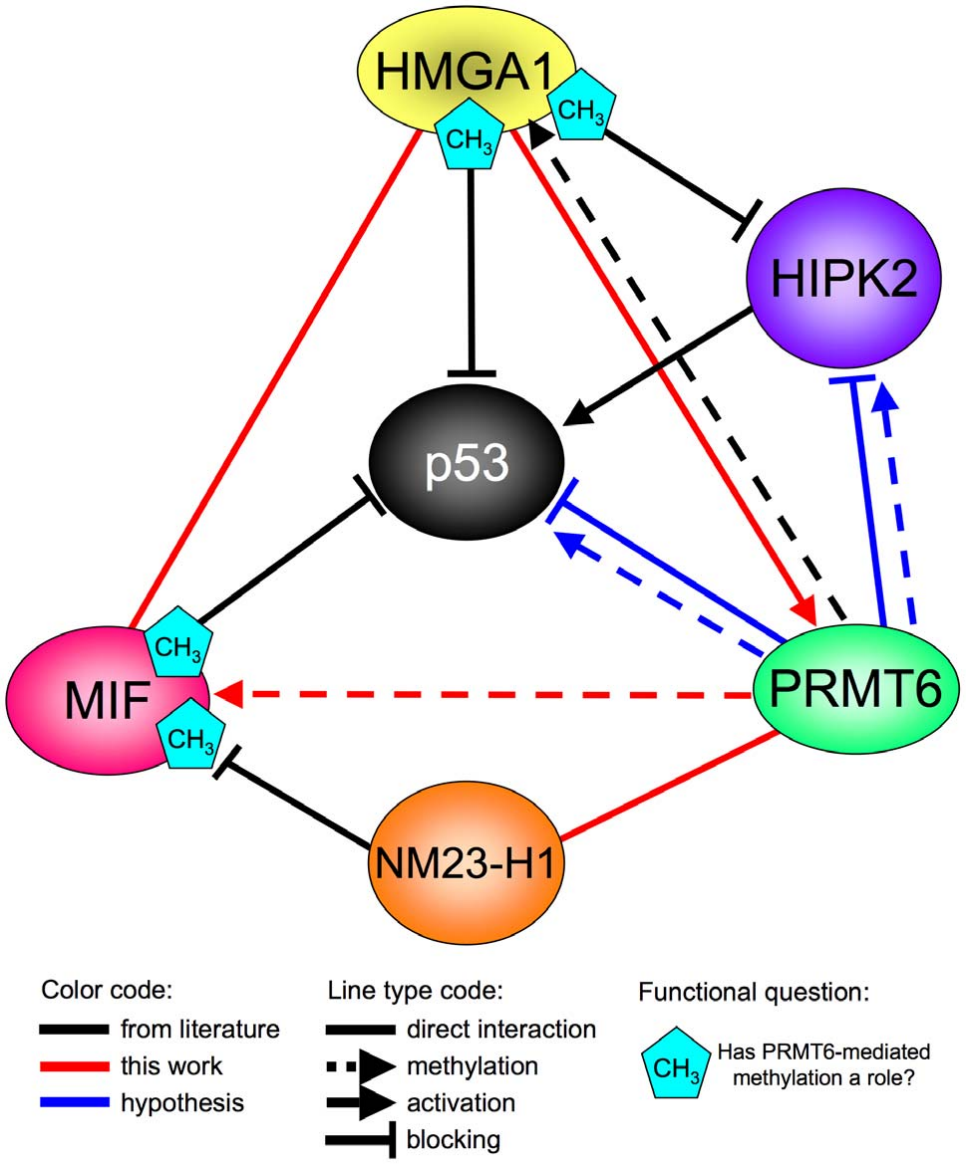
From interaction data to functional hypothesis. Schematic representation of the molecular network impinging on p53 and directly linked to PRMT6. The modulatory effects reported in this scheme (activation or blocking) are exclusively mediated by direct protein-protein interactions. The methyltransferase activity of PRMT6 toward its substrates implies a direct protein-protein contact.

In summary:

1. with our Y2H approach we were able to discover 36 new molecular partners for PRMT6. The large majority of PRMT6 interactors tested resulted to be confirmed by our *in vitro* and *in vivo* protein-protein interaction experiments. Therefore, Y2H resulted a reliable approach to fish out bona-fide cellular partners of PRMT6. Moreover, 4 new substrates for PRMT6 were discovered (see Table 1 for a summary of these results). The identification of new partners and substrates for PRMT6 and their characteristics suggests a wide involvement of PRMT6 in the context of cell biology. A clear limitation of our study is that it is mainly based on overexpression of proteins in fusion with tags and therefore this makes difficult to assess how relevant are the interactions found *in vivo*. Once focused on selected partners/substrates other approaches should be considered to assess whether they are real PRMT6 targets. Reciprocal Co-IP experiments should be performed with the endogenous proteins and these data should be supported by orthogonal strategies, such as *in vivo* co-localization imaging (co-immunolocalization and/or FRET). In addition, the PRMT6-dependent methylation status of these proteins should be assessed after PRMT6 silencing/knock-out and mapped *in vivo*. Nevertheless, our data constitute the basis for future hypothesis-driven investigations;
2. considering that proteins in close proximity within protein-protein interaction networks have a strong probability to interact to each other and that PRMT6 and HMGA are direct molecular partners, we were able to discover new molecular partners for HMGA proteins among the group of proteins initially isolated in this work as PRMT6 interactors;
3. we provided a proof of concept that the close proximity within protein-protein interaction networks could underline a functional link. Indeed, MIF methylation by PRMT6 turned out to be, at least *in vitro*, modulated by the presence of HMGA1a. Our Y2H approach revealed a network composed by HMGA1a, PRMT6, MIF, and NM23-H1, which are all directly or indirectly linked to p53 activity modulation. Noteworthy, our results assume relevance in light of the very recent findings demonstrating a direct transcriptional modulatory role of PRMT6 with respect to p53 and its direct target p21 [19], [20], [22], suggesting a potential additional layer of modulatory mechanisms impinging on the p53 node. Therefore, this network could be used as a framework to build up functional hypotheses (see Fig. 6) to be subsequently experimentally validated.

**Table 1:** Y2H 1^st^: yeast two-hybrid screening.

Table 1: Summary of protein-protein interaction data and enzymatic assays						
Y2H		GST-pull down			Co-AP	
1^st^	2^nd^	PRMT6	HMGA1	HMGA2	PRMT6	Methylation
Med28	Med28	n.a	n.a	n.a	n.s	n.s
MTF2	MTF2	n.a	n.a	n.a	MTF2	n.s
CDK5RAP3	CDK5RAP3	CDK5RAP3	neg.	neg.	n.s	n.s
Nm23-H1	Nm23-H1^*^	neg.	neg.	neg.	Nm23-H1	neg.
EBP1	EBP1	EBP1	neg.	neg.	n.s	n.s
NOB1	NOB1	NOB1	neg.	NOB1	NOB1	neg.
UTP6	UTP6	n.a	n.a	n.a	n.s	n.s
hnRNP Q	hnRNP Q^#^	hnRNP Q	hnRNP Q	hnRNP Q	neg.	n.s
GRSF-1	GRSF-1	neg.	neg.	neg.	n.s	n.s
CDK9	CDK9	CDK9	neg.	neg.	n.s	n.s
snRNPB	snRNPB	snRNPB	snRNPB	snRNPB	n.s	snRNPB
PRPF39	PRPF39	PRPF39	PRPF39	PRPF39	n.s	n.s
PSMD11	n.s	n.s	n.s	n.s	n.s	n.s
PSME1	n.s	n.s	n.s	n.s	n.s	n.s
PSMB4	n.s	n.s	n.s	n.s	n.s	n.s
POMP	n.s	n.s	n.s	n.s	n.s	n.s
HYPK	HYPK^#^	neg.	neg.	neg.	n.s	n.s
PRDX4	PRDX4	n.a.	n.a.	n.a	n.s	n.s
SAAL1	SAAL1	n.a.	n.a.	n.a	n.s	n.s
FtL	FtL	FtL	neg.	neg.	n.s	n.s
HSPB1	n.s	n.s	n.s	n.s	n.s	n.s
MIF	MIF	MIF	MIF	MIF	neg.	MIF
Hint1	Hint1	Hint1	neg.	neg.	n.s	n.s
HPRT1	HPRT1	HPRT1	neg.	neg.	n.s	n.s
MRPL38	MRPL38^*^	n.a	n.a	n.a	n.s	n.s
LDHB	LDHB^§^	n.a	n.a	n.a	n.s	n.s
FH	FH	FH	neg.	neg.	n.s	n.s
PTS	PTS	PTS	PTS	PTS	PTS	n.s
QPRT	QPRT	neg.	neg.	neg.	n.s	n.s
COPS3	COPS3	COPS3	COPS3	COPS3	n.s	n.s
PRKX	PRKX	n.a	n.a	n.a	n.s	n.s
CASP6	CASP6^#^	CASP6	CASP6	CASP6	CASP6	n.s
SVEP1	SVEP1	SVEP1	SVEP1	SVEP1	n.s	neg.
TUBB2A	TUBB2A	TUBB2A	neg.	neg.	TUBB2A	TUBB2A
SEPT7	SEPT7	SEPT7	neg.	neg.	n.s	n.s
HSJ-2	HSJ-2	HSJ-2	HSJ-2	HSJ-2	HSJ-2	HSJ-2

Y2H 2^nd^: Mapping of the interacting domain. Proteins interacting with FL and 1–184 forms are marked with #, those interacting with FL and 1–86 forms are marked with *, that interacting only with FL PRMT6 is marked with §, and those interacting with FL, 1–86, and 1–184 PRMT6 forms are left unmarked. neg.: negative assays. n.s: not selected. n.a: not available.

## Supporting Information

Table S1 **List of PRMT6-interacting proteins identified by yeast two-hybrid screening.**
(DOC)

Table S2 **Gene ontology (GO) annotation of PRMT6 interactors.**
(DOC)

Table S3 **Mapping of the interacting domain of PRMT6.**
(DOC)

Materials and Methods S1 **Supporting materials and methods.**
(DOC)
