# Correction: Identification and Characterization of New Molecular Partners for the Protein Arginine Methyltransferase 6 (PRMT6)

**DOI:** 10.1371/annotation/37947ebc-7ddb-4500-8441-8a4804b0fb5e

**Published:** 2013-11-08

**Authors:** Alessandra Lo Sardo, Sandro Altamura, Silvia Pegoraro, Elisa Maurizio, Riccardo Sgarra, Guidalberto Manfioletti

The authors would like to provide some clarification in relation to Figures 1B, 4A, 4B and 5A in the article.

Each of the figures was generated using lanes that originate from two separate gels, we are providing revised figure files where the lanes that originate from different gels are separated by vertical lines.

Figure 1B. Figure 1B derives from two gels exposed together. The vertical line between lane 3 and 4 indicates that two lanes were not loaded and therefore in the figure they were spliced out. The line between lane 6 and 7 is instead due to the assembly of the two different blots.

Figure 4A and B. Figure 4 derives from two gels. The vertical line between lanes 7 and 8 indicates that two fluorography (Figure 4A) and the corresponding Blue Comassie stained gels (Figure 4B) were assembled together.

Figure 5A. Figure 5A derives from two gels exposed together. The vertical line between lanes 7 and 8 indicates the assembly of the fluorography of the two gels.

Figure 1: 

**Figure:**
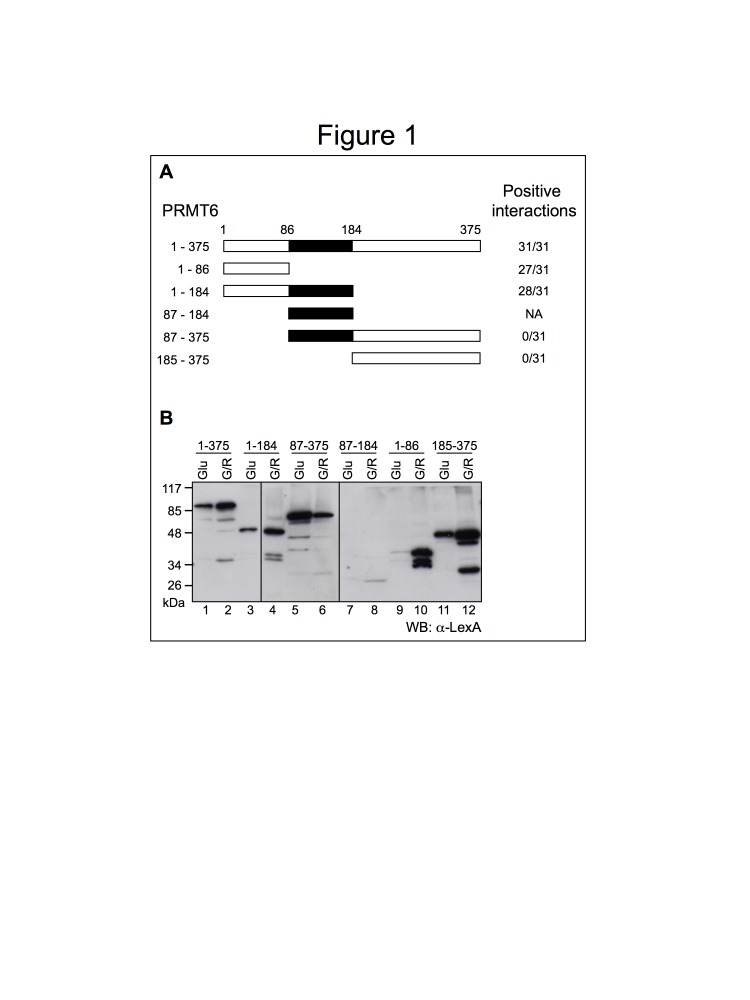


Figure 4: 

**Figure:**
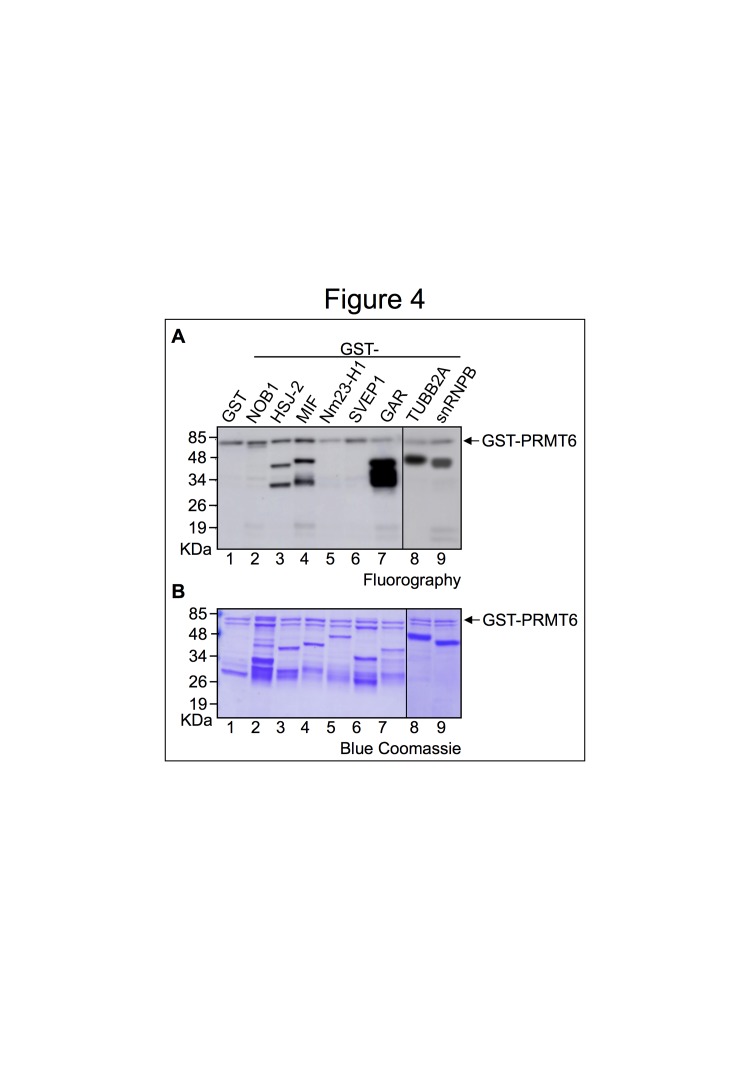


Figure 5: 

**Figure:**
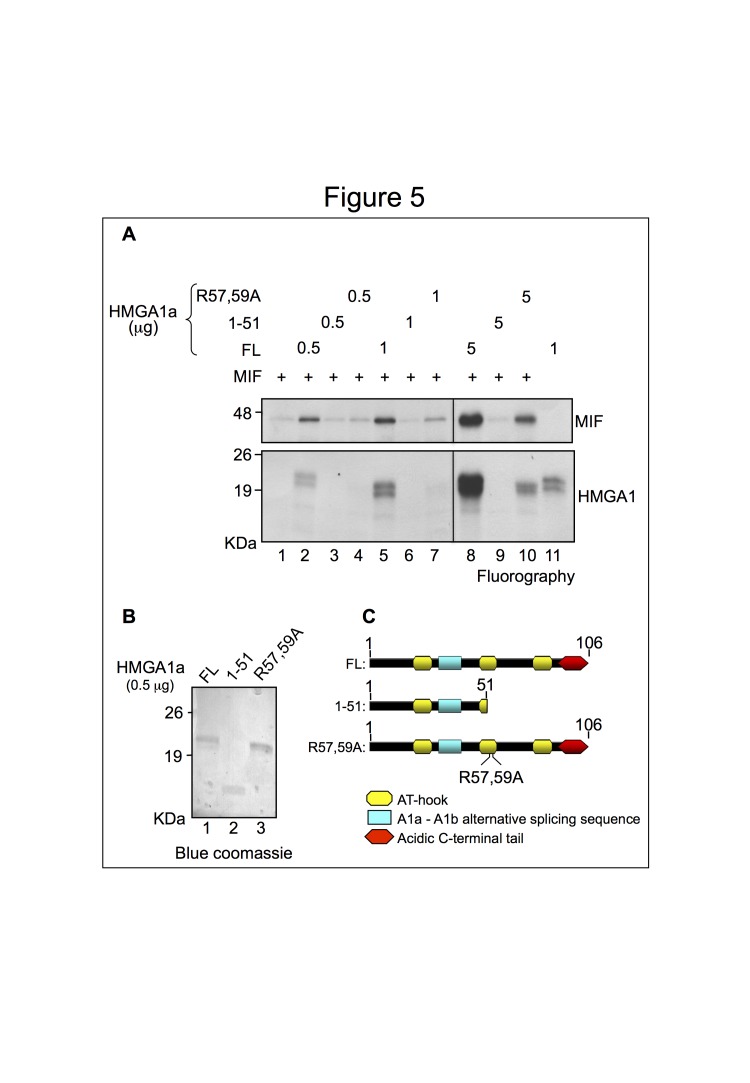


Original blot for Figure 1B: http://www.plosone.org/corrections/pone.0053750.g001B.cn.tif

Original blot for Figure 4A, left panel: http://www.plosone.org/corrections/pone.0053750.g004AL.cn.tif

Original blot for Figure 4A, right panel: http://www.plosone.org/corrections/pone.0053750.g004AR.cn.tif

Original blot for Figure 4B, left panel: http://www.plosone.org/corrections/pone.0053750.g004BL.cn.tif

Original blot for Figure 4B, right panel: http://www.plosone.org/corrections/pone.0053750.g004BR.cn.tif

Original blot for Figure 5A: http://www.plosone.org/corrections/pone.0053750.g005A.cn.tif

