# Supplementary material for: Identification and Characterization of New Molecular Partners for the Protein Arginine Methyltransferase 6 (PRMT6)
Source: PLoS One. 2013 Jan 10;8(1):e53750. doi: 10.1371/journal.pone.0053750 (PMC3542376; doi:10.1371/journal.pone.0053750)
Supplement: Table S1 — List of PRMT6-interacting proteins identified by yeast two-hybrid screening. (DOC) [file pone.0053750.s001.doc]

**Table S1.** **List of PRMT6-interacting proteins identified by yeast two-hybrid screening.** For each partner is shown the length of the encoded protein identified with respect to the full length, the function, the number of clones found with the Y2H and the Uniprot accession number.

| **Protein interacting with PRMT6** | **Coded prot. residues** | **Function** | **Found clones** | **Accession**  **number** |
| --- | --- | --- | --- | --- |
| Mediator of RNA polymerase II transcription subunit 28 (Med28, Magicin) | 2-178/178 | Component of the Mediator complex which regulate transcription of nearly all RNA polymerase II-dependent genes. | 71 | Q9H204 |
| Metal-response element-binding transcription factor 2, Polycomb-like protein 2 (MTF2, PCL2) | 387-586/586 | Binds to the metal-regulating-element (MRE) of metallothionein-1A gene promoter. It is part of the polycomb repressive complex-2 (PRC2). | 1 | Q9Y483 |
| CDK5 regulatory subunit-associated protein 3 (CDK5RAP3) | 395-506/506 | Potential regulator of CDK5 activity. May be involved in cell proliferation. | 1 | Q96JB5 |
| Nucleoside diphosphate kinase A (Nm23-H1) | 1-152/152 | Major role in the synthesis of nucleoside triphosphates other than ATP. Involved in cell proliferation, differentiation and development, signal transduction, G protein-coupled receptor endocytosis, and gene expression. | 2 | P15531 |
| Proliferation-associated protein 2G4 (EBP1) | 155-394/394 | May play a role in a ERBB3-regulated signal transduction pathway. Seems be involved in growth regulation. | 3 | Q9UQ80 |
| RNA-binding protein NOB1 | 134-412/412 | May play a role in mRNA degradation. | 2 | Q9ULX3 |
| U3 small nucleolar RNA-associated protein 6 homolog (UTP6) | 434-597/597 | Nucleolar processing of pre-18S ribosomal RNA. | 1 | Q9NYH9 |
| Heterogeneous nuclear ribonucleoprotein Q (hnRNP Q) | 217-410/410 | mRNA processing mechanisms. | 1 | O60506 |
| G-rich sequence factor 1 (GRSF-1) | 250-480/480 | Binds RNAs containing the 14 base G-rich element. | 2 | Q12849 |
| Cyclin-dependent kinase 9 (CDK9) | 250-372/372 | Member of the cyclin-dependent kinase pair (CDK9/cyclin-T) complex which facilitates the transition from abortive to production elongation by phosphorylating the CTD (C-terminal domain) of the large subunit of RNA polymerase II. | 1 | P50750 |
| Small nuclear ribonucleoprotein-associated proteins B and B' (snRNPB) | 145-231/231 | May have a functional role in the pre-mRNA splicing or in snRNP structure. Appears to function in the U7 snRNP complex that is involved in histone 3'-end processing. | 1 | P14678 |
| Pre-mRNA-processing factor 39 (PRPF39) | 485-669/669 | Involved in pre-mRNA splicing. | 1 | Q86UA1 |
| 26S proteasome non-ATPase regulatory subunit 11 (PSMD11) | 74-422/422 | Acts as a regulatory subunit of the 26S proteasome which is involved in the ATP-dependent degradation of ubiquitinated proteins. | 10 | O00231 |
| Proteasome activator complex subunit 1 (PSME1) | 1-249/249 | Implicated in immunoproteasome assembly and required for efficient antigen processing. | 2 | Q06323 |
| Proteasome subunit beta type-4 (PSMB4) | 1-264/264 | Subunit of the proteasome, multicatalytic proteinase complex. | 1 | P28070 |
| Proteasome maturation protein (POMP) | 1-141/141 | Chaperone essential for the assembly of standard proteasomes and immunoproteasomes. | 1 | Q9Y244 |
| Huntingtin-interacting protein K (HYPK) | 10-129/129 | May interact with the N-terminus of HD. | 17 | Q9NX55 |
| Peroxiredoxin-4 (PRDX4) | 15-271/271 | Probably involved in redox regulation of the cell. Regulates the activation of NF-kappa-B in the cytosol. | 5 | Q13162 |
| Protein SAAL1 (SAAL1) | 1-474/474 | Belongs to the SAAL1 family. Unknown function. | 5 | Q96ER3 |
| Ferritin L-chain (FtL) | 1-175/175 | Storage of iron in a soluble and nontoxic state. | 1 | P02792 |
| Heat shock protein beta-1 (HSPB1) | 1-205/205 | Involved in stress resistance and actin organization. | 1 | Q96EI7 |
| Macrophage migration inhibitory factor (MIF) | 34-115/115 | Pro-inflammatory cytokine. Suggested mediator in regulating the function of macrophages in host defense. | 8 | P14174 |
| Histidine triad nucleotide-binding protein 1 (Hint1) | 1-126/126 | Hydrolyzes adenosine 5'monophosphoramidate substrates. | 6 | P49773 |
| Hypoxanthine-guanine phosphoribosyltransferase (HPRT1) | 1-218/218 | Plays a central role in the generation of purine nucleotides through the purine salvage pathway. Defects in HPRT1 are the cause of Lesch-Nyhan syndrome (LNS). | 9 | P00492 |
| 39S ribosomal protein L38 (MRPL38) | 46-346/346 | Ribosomal protein. | 2 | Q96Q66 |
| L-lactate dehydrogenase B chain (LDHB) | 140-334/334 | Metabolic enzyme. | 2 | P07195 |
| Fumarate hydratase, mitochondrial (FH) | 188-510/510 | Carbohydrate metabolism. Also acts as a tumor suppressor. | 1 | P07954 |
| 6-pyruvoyl tetrahydrobiopterin synthase, (PTS) | 1-145/145 | Involved in the biosynthesis of tetrahydrobiopterin. | 4 | Q03393 |
| Nicotinate-nucleotide pyrophosphorylase (QPRT) | 184-296/296 | Involved in the catabolism of quinolinate, an intermediate in the tryptophan-nicotinamide adenine dinucleotide pathway. | 7 | Q15274 |
| COP9 signalosome complex subunit 3 (COPS3) | 116-423/423 | Involved in various cellular and developmental processes. | 1 | Q9UNS2 |
| Serine/threonine-protein kinase PRKX (PRKX) | 283-402/402 | Belong to the protein kinase superfamily. AGC Ser/Thr protein kinase family. cAMP subfamily. A chromosomal aberration involving PRKX is a cause of sex reversal disorder. | 1 | P51817 |
| Caspase-6 (CASP6) | 1-293/293 | Involved in the activation cascade of caspases responsible for apoptosis execution. Overexpression promotes programmed cell death. | 2 | P55212 |
| Sushi, von Willebrand factor type A (SVEP1) | 3464-3571/3571 | May play a role in the cell attachment process. | 3 | Q4LDE5 |
| Tubulin beta-2A chain (TUBB2A) | 284-445/445 | Tubulin is the major constituent of microtubules. It binds two moles of GTP, one at an exchangeable site on the beta chain and one at a non-exchangeable site on the alpha-chain. | 1 | Q13885 |
| Septin-7 (SEPT7) | 20-437/437 | Filament-forming cytoskeletal GTPase. Required for normal organization of the actin cytoskeleton. Required for normal progress through mitosis. Involved in cytokinesis. | 1 | Q16181 |
| DnaJ homolog subfamily B member 6, Heat Shock protein J-2 (DNAJB6, HSJ-2) | 121-241/241 | Acts as an endogenous molecular chaperone inhibiting the Wnt/beta-catenin pathway by inducing beta-catenin degradation. | 1 | O75190 |
