# Supplementary material for: Identification and Characterization of New Molecular Partners for the Protein Arginine Methyltransferase 6 (PRMT6)
Source: PLoS One. 2013 Jan 10;8(1):e53750. doi: 10.1371/journal.pone.0053750 (PMC3542376; doi:10.1371/journal.pone.0053750)
Supplement: Table S2 — Gene ontology (GO) annotation of PRMT6 interactors. (DOC) [file pone.0053750.s002.doc]

**Table S2. Gene ontology (GO) annotation of PRMT6 interactors.**

The Table lists significantly (p<0.05) enriched GO terms.

| **ENRICHED FUNCTIONAL ANNOTATION**  **BIOLOGICAL PROCESS** | **PValue** | **Genes** |
| --- | --- | --- |
| GO:0006461~protein complex assembly | 3.8E-3 | TUBB2A, CDK9, QPRT, HPRT1, SEPT7, MIF |
| GO:0006396~RNA processing | 5.3E-3 | PA2G4, UTP6, SNRPB, GRSF1, SYNCRIP, PRPF39, PA2G4P4 |
| GO:0051259~protein oligomerization | 5.8E-3 | QPRT, HPRT1, SEPT7, MIF |
| GO:0051436~negative regulation of ubiquitin-protein ligase activity during mitotic cell cycle | 8.3E-3 | PSMB4, PSME1, PSMD11 |
| GO:0051437~positive regulation of ubiquitin-protein ligase activity during mitotic cell cycle | 9.1E-3 | PSMB4, PSME1, PSMD11 |
| GO:0051439~regulation of ubiquitin-protein ligase activity during mitotic cell cycle | 9.8E-3 | PSMB4, PSME1, PSMD11 |
| GO:0016070~RNA metabolic process | 1.2E-2 | PA2G4, UTP6, SNRPB, GRSF1, SYNCRIP, PRPF39, CDK9, PA2G4P4 |
| GO:0051340~regulation of ligase activity | 1.2E-2 | PSMB4, PSME1, PSMD11 |
| GO:0032268~regulation of cellular protein metabolic process | 1.7E-2 | PSMB4, PA2G4, PSME1, PSMD11, HSPBL2, HSPB1, PA2G4P4 |
| GO:0043086~negative regulation of catalytic activity | 2.0E-2 | PSMB4, PSME1, PSMD11, DNAJB6 |
| GO:0051246~regulation of protein metabolic process | 2.7E-2 | PSMB4, PA2G4, PSME1, PSMD11, HSPBL2, HSPB1, PA2G4P4 |
| GO:0043436~oxoacid metabolic process | 2.8E-2 | LDHB, QPRT, PTS, FH, MIF |
| GO:0055086~nucleobase, nucleoside and nucleotide metabolic process | 2.8E-2 | LDHB, NME1-NME2, QPRT, HPRT1 |
| GO:0044271~nitrogen compound biosynthetic process | 3.1E-2 | NME1-NME2, QPRT, HPRT1, PTS |
| GO:0006732~coenzyme metabolic process | 4.1E-2 | LDHB, QPRT, FH |
|  |  |  |
| **ENRICHED FUNCTIONAL ANNOTATION:**  **CELLULAR COMPARTMENT** | **PValue** | **Genes** |
| GO:0000502~proteasome complex | 6.8E-6 | PSMB4, PSME1, PSMD11, HSPBL2, POMP, HSPB1 |
| GO:0044424~intracellular part | 6.2E-5 | LDHB, TUBB2A, COPS3, HINT1, HSPBL2, UTP6, PRDX4, NOB1, SYNCRIP, HPRT1, MIF, PSMB4, CASP6, MED28, LOC100132364, MRPL38, PTS, FH, SVEP1, GRSF1, CDK9, PRPF39, PA2G4, PSME1, NME1-NME2, MTF2, PSMD11, SNRPB, POMP, HSPB1, QPRT, SEPT7, PA2G4P4, DNAJB6 |
| GO:0005737~cytoplasm | 6.8E-4 | LDHB, COPS3, HINT1, HSPBL2, PRDX4, SYNCRIP, HPRT1, MIF, CASP6, PSMB4, MED28, MRPL38, PTS, FH, SVEP1, GRSF1, PA2G4, PSME1, NME1-NME2, PSMD11, SNRPB, POMP, HSPB1, QPRT, SEPT7, DNAJB6, PA2G4P4 |
| GO:0005634~nucleus | 1.1E-2 | COPS3, HINT1, HSPBL2, UTP6, NOB1, SYNCRIP, CDK9, PRPF39, CASP6, PSMB4, PA2G4, NME1-NME2, MED28, MTF2, POMP, SNRPB, HSPB1, LOC100132364, SEPT7, DNAJB6, PA2G4P4 |
| GO:0005829~cytosol | 1.5E-2 | CASP6, PSME1, NME1-NME2, PSMD11, POMP, SNRPB, QPRT, HPRT1 |
| GO:0044444~cytoplasmic part | 1.8E-2 | HSPBL2, PRDX4, SYNCRIP, HPRT1, CASP6, PSMB4, PSME1, NME1-NME2, PSMD11, POMP, SNRPB, HSPB1, MRPL38, QPRT, PTS, SEPT7, DNAJB6, FH |
| GO:0005625~soluble fraction | 2.5E-2 | LDHB, NME1-NME2, HSPBL2, HSPB1, HPRT1 |
| GO:0043232~intracellular non-membrane-bounded organelle | 2.8E-2 | TUBB2A, HINT1, UTP6, HSPBL2, CDK9, PRPF39, PSMB4, PA2G4, NME1-NME2, HSPB1, MRPL38, SEPT7, PA2G4P4 |
