# Supplementary material for: Identification and Characterization of New Molecular Partners for the Protein Arginine Methyltransferase 6 (PRMT6)
Source: PLoS One. 2013 Jan 10;8(1):e53750. doi: 10.1371/journal.pone.0053750 (PMC3542376; doi:10.1371/journal.pone.0053750)
Supplement: Table S3 — Mapping of the interacting domain of PRMT6. (DOC) [file pone.0053750.s003.doc]

**Table S3.** **Mapping of the interacting domain.** Each partner was tested in two-hybrid assay for its ability to bind 5 different deletion mutants. Mutant PRMT6 87-184 could not be used because it was not express in yeast, and it is reported as NA (Not Available). The + indicates the interaction. PRMT6 and ODC were added as a positive and negative control respectively.

| **PARTNERS** | **PRMT6** | | | | | |
| --- | --- | --- | --- | --- | --- | --- |
|  | **Full lenght** | **1-86** | **1-184** | **87-184** | **87-375** | **185-375** |
| Med28 | + | + | + | NA | - | - |
| MTF2 | + | + | + | NA | - | - |
| CDK5RAP3 | + | + | + | NA | - | - |
| Nm23-H1 | + | + | - | NA | - | - |
| EBP1 | + | + | + | NA | - | - |
| NOB1 | + | + | + | NA | - | - |
| UTP6 | + | + | + | NA | - | - |
| hnRNP Q | + | - | + | NA | - | - |
| GRSF-1 | + | + | + | NA | - | - |
| CDK9 | + | + | + | NA | - | - |
| SnRNP-B | + | + | + | NA | - | - |
| PRPF39 | + | + | + | NA | - | - |
| HYPK | + | - | + | NA | - | - |
| PRDX4 | + | + | + | NA | - | - |
| SAAL1 | + | + | + | NA | - | - |
| FtL | + | + | + | NA | - | - |
| MIF | + | + | + | NA | - | - |
| HINT1 | + | + | + | NA | - | - |
| HPRT1 | + | + | + | NA | - | - |
| L38mt | + | + | - | NA | - | - |
| LDH-B | + | - | - | NA | - | - |
| FH | + | + | + | NA | - | - |
| PTPS | + | + | + | NA | - | - |
| QPRTase | + | + | + | NA | - | - |
| COPS3 | + | + | + | NA | - | - |
| PRKX | + | + | + | NA | - | - |
| CASP6 | + | - | + | NA | - | - |
| SVEP1 | + | + | + | NA | - | - |
| TUBB2A | + | + | + | NA | - | - |
| SEPT7 | + | + | + | NA | - | - |
| HSJ-2 | + | + | + | NA | - | - |
| PRMT6 | + | + | + | NA | - | - |
| ODC | - | - | - | NA | - | - |
