# Supplementary material for: Identification and Characterization of New Molecular Partners for the Protein Arginine Methyltransferase 6 (PRMT6)
Source: PLoS One. 2013 Jan 10;8(1):e53750. doi: 10.1371/journal.pone.0053750 (PMC3542376; doi:10.1371/journal.pone.0053750)
Supplement: Materials and Methods S1 — Supporting materials and methods. (DOC) [file pone.0053750.s004.doc]

**Materials and Methods S1**

Plasmids pEG202 PRMT6 1-86, pEG202 PRMT6 1-184, pEG202 PRMT6 87-184, pEG202 PRMT6 87-375, pEG202 185-375 expressing deletion mutants of human PRMT6 protein were generated by PCR using the human PRMT6 cDNA as a template. The primers used for the construction were as follows: PRMT6 wt up, 5’-TTC GGG AAT TCA TGT CGC AGC CCA A-3’; PRMT6 wt dw, 5’-TTC AAA GTC GAC TCA GTC CTC CAT GGC-3’; PRMT6 184 dw, 5’-TTC AAA GTC GAC GAG AAG ACC GCC C-3’; PRMT6 Δ86 up, 5’-AAT ATT GAA TTC CTG GAC GTG GGC GCG-3’; PRMT6 86 dw, 5’-ATT CGT CGA CTC ATA CCG TCT TGC CTC G-3’; PRMT6 Δ184 up, 5’-AAA GAA TTC CTG CCG GCC TCC G-3’. The PCR products for the deletion mutants were obtained using the appropriate combination of upstream (up) and downstream (dw) primers. The PCR products were cloned between *Eco*RI and *Sal*I sites of the pEG202 vector. The resulting clones were verified by sequencing. The cDNA for PRDX4, HPRT1, and PKX1 were cloned in pcDNA3HA vector and in pGST4T-1 by PCR using as template the corresponding cDNAs in pJG4-5 using the following primers: PRDX4 up, 5’-AAT TAA GGA TCC GCC ACC GAA GGC TGC-3’; PRDX4 dw, 5’-GGT GGC TCG AGG GTA ATG AAA CCG TGA AC-3’; HPRT1 up, 5’-GGC GGG GAT CCG GGG CTA TAA ATT CTT-3’; HPRT1 dw, 5’-GGC GGG TCT AGA ATA GTG CTG TGG GTT AA-3’; PKX1 up, 5’-AAA TAA GGA TCC CCC CGG ACT GGG CG-3’; PKX1 dw, 5’-AAG GTC TCG AGT CAG AAT CTG GCA GAT GAC-3’. The cDNA for GRSF, MTF2, SnRNP B, CDK5, and Septin 7 were cloned in two steps in pcDNA3HA between the enzymes *Bam*HI-*Xho*I (GRSF), *Bam*HI-*Xho*I (MTF2), *Eco*RI-*Xba*I (SnRNP B), *Bam*HI-*Xba*I (CDK5), and *Bam*HI, *Xba*I (Septin 7).
